# Large-scale association study on daily weight gain in pigs reveals overlap of genetic factors for growth in humans

**DOI:** 10.1186/s12864-022-08373-3

**Published:** 2022-02-15

**Authors:** Zexi Cai, Ole Fredslund Christensen, Mogens Sandø Lund, Tage Ostersen, Goutam Sahana

**Affiliations:** 1 Center for Quantitative Genetics and Genomics, Aarhus University, 8830 Tjele, Denmark; 2 SEGES Danish Pig Research Centre, Agro Food Park 15, 8200 Aarhus N, Denmark

**Keywords:** GWAS, Large-scale association study, Pig breeding, Imputation, Candidate genes

## Abstract

**Background:**

Imputation from genotyping array to whole-genome sequence variants using resequencing of representative reference populations enhances our ability to map genetic factors affecting complex phenotypes in livestock species. The accumulation of knowledge about gene function in human and laboratory animals can provide substantial advantage for genomic research in livestock species.

**Results:**

In this study, 201,388 pigs from three commercial Danish breeds genotyped with low to medium (8.5k to 70k) SNP arrays were imputed to whole genome sequence variants using a two-step approach. Both imputation steps achieved high accuracies, and in total this yielded 26,447,434 markers on 18 autosomes. The average estimated imputation accuracy of markers with minor allele frequency ≥ 0.05 was 0.94. To overcome the memory consumption of running genome-wide association study (GWAS) for each breed, we performed within-breed subpopulation GWAS then within-breed meta-analysis for average daily weight gain (ADG), followed by a multi-breed meta-analysis of GWAS summary statistics. We identified 15 quantitative trait loci (QTL). Our post-GWAS analysis strategy to prioritize of candidate genes including information like gene ontology, mammalian phenotype database, differential expression gene analysis of high and low feed efficiency pig and human GWAS catalog for height, obesity, and body mass index, we proposed *MRAP2*, *LEPROT*, PMAIP1, *ENSSSCG00000036234, BMP2, ELFN1, LIG4* and *FAM155A* as the candidate genes with biological support for ADG in pigs.

**Conclusion:**

Our post-GWAS analysis strategy helped to identify candidate genes not just by distance to the lead SNP but also by multiple sources of biological evidence. Besides, the identified QTL overlap with genes which are known for their association with human growth-related traits. The GWAS with this large data set showed the power to map the genetic factors associated with ADG in pigs and have added to our understanding of the genetics of growth across mammalian species.

**Supplementary Information:**

The online version contains supplementary material available at 10.1186/s12864-022-08373-3.

## Background

The number of genome-wide association studies (GWAS) has grown rapidly over the last decade to establish a link between genetic variants and complex traits in humans and agricultural species. Genotype data for GWAS are usually generated using cost-effective genotyping arrays of common single nucleotide polymorphism (SNP) variants. The use of imputed whole-genome sequencing (WGS) is routine in human GWAS [1] due to the availability of whole-genome haplotype reference panels. A similar reference panel for cattle is available and routinely used in cattle GWAS studies [2, 3]. However, the imputation of a SNP array to WGS for GWAS in pigs is still not common. One reason could be the absence of an international haplotype panel in pigs. Imputation from a low-density marker set to a high-density marker set, and even up to WGS level, has shown high accuracy at an affordable cost for large-scale GWAS [3–6] and investigation of the genetic architecture of complex traits [7–9]. Normally, the reference panel for imputation requires a large number of individuals. However, the whole genome sequencing of many animals is still economically prohibitive. A previous study [10] showed the advantage of a two-step imputation strategy in cattle, where step 1: impute a low-density (50k) SNP array marker set to a high-density (700k) SNP array marker set; and step 2: impute the imputed high-density marker set to WGS. Brøndum et al. [11] showed that a multi-breed reference panel can increase imputation accuracy in cattle. Both strategies, namely the two-step imputation and multi-breed reference population, can also be used to increase imputation accuracy in other livestock species like pigs. Unlike pure breeding in dairy cattle, two-way and three-way crosses are routinely used to produce slaughter pigs. Although we know that multi-breed reference could improve imputation accuracy, it is worth examining whether available high-density (HD) genotypes from crossbred pigs can be used as the intermediate reference panel for purebred pigs.

The growth rate is an important trait in pig breeding, as it is directly linked to economic returns. Average daily gain (ADG) is one of the most important indicators of the growth rate and indicates the time required for pigs to achieve the targeted market weight [12]. Previous studies have shown the complex genetic architecture of ADG [13], which puts a limit on how precisely the quantitative trait loci (QTL) can be determined. Approximately 753 QTL for ADG spread across all chromosomes in pigs are reported in the QTL database (queried in July 2020) [14]. Recently, Falker-Gieske et al. performed GAWS using imputed WGS to identify QTLs on chromosome 2, 4 and 7 for ADG [15]. Similarly, a larger number of genetic factors affecting growth-related traits in humans and other mammalian species are known [3, 16, 17]. Therefore, precise mapping of genetic factors for ADG in pigs could highlight the common genetic factors affecting growth in humans and other mammalian species. Furthermore, in pig breeding, mapped WGS variants, if included in a genomic selection marker panel, may increase the prediction accuracy for ADG [18].

The knowledge gained from non-human species could bring new insights for human studies. Mice are one common model species for human research [19, 20], but the scale of species could expand to other rodent species [21]. Researches have used model species to study specific human diseases, e.g., ferrets, as a model for human respiratory disease [22], and sheep as a model for human asthma and other respiratory diseases [23]. Similarly, for quantitative traits, meta-analysis of cattle stature revealed the genetic similarity between human height and cattle stature [3]. A pig model is used to study several human traits [24–27]. Therefore, the mapping of genetic factors for ADG in pigs could be utilized to add to our knowledge about growth traits in humans.

The aim of this study was to detect the WGS variants associated with ADG in pigs, and to study whether the candidate genes underlying these associated variants in pigs for ADG are known for their association with growth-related traits in humans. To achieve this goal, we used phenotypes and SNP array genotypes from 201,388 animals from three Danish pig breeds. We divided each breed into subpopulations to run GWAS and then carried out a within-breed meta-analysis, followed by a multi-breed meta-analysis. In the post-GWAS analyses, we examined whether the identified candidate genes in pigs are known to be related to growth-related phenotypes in humans.

## Results

### Imputation to WGS level

**Fig. 1:**
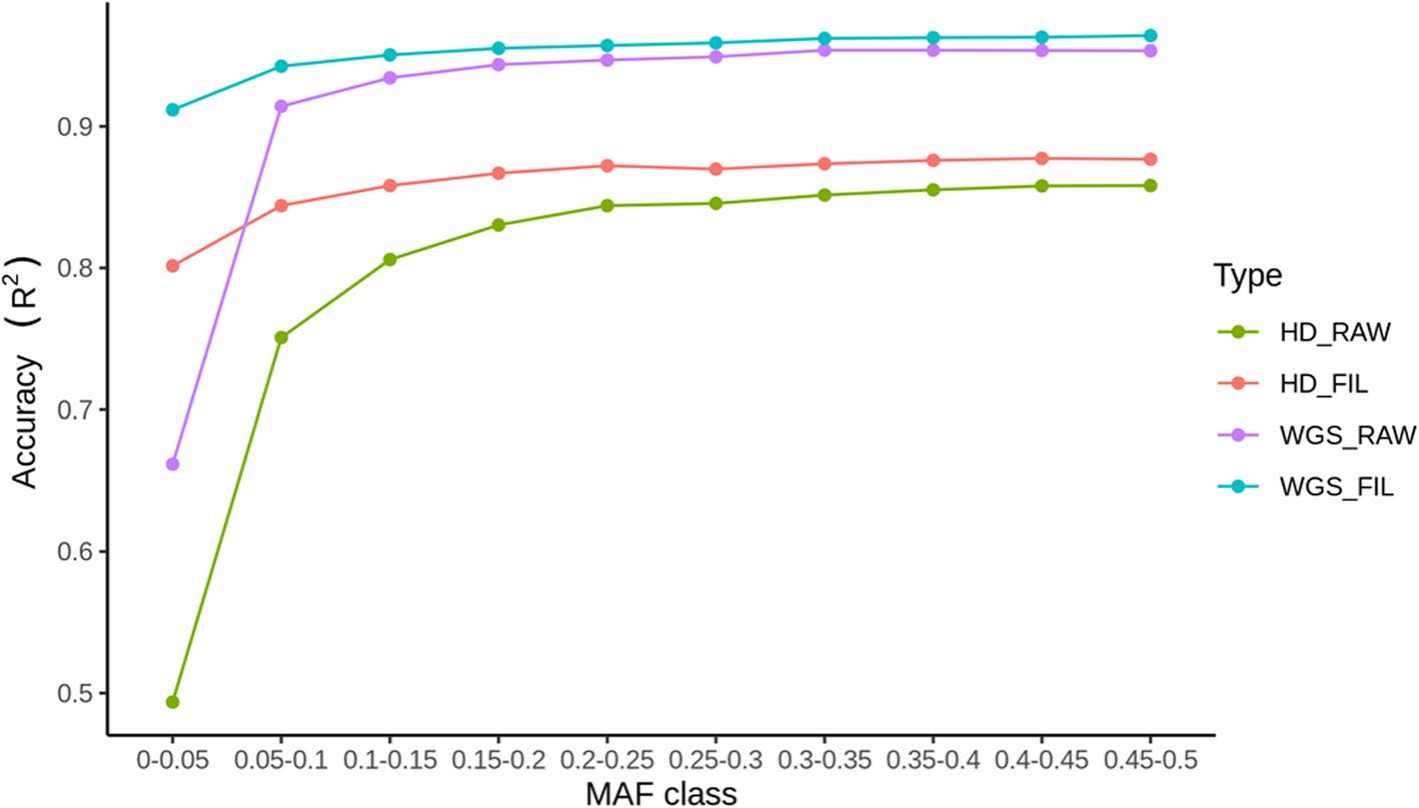
Average estimated imputation accuracy across different minor allele frequencies (MAF); HD_RAW is for imputation from low-density chip to HD (all markers); HD_FIL is for imputation from 60 k to HD for markers with estimated imputation accuracy ≥ 0.4; WGS_RAW is for imputation from imputed HD to WGS (all markers); and WGS_FIL is for imputation from imputed HD to WGS for markers with estimated imputation accuracy ≥ 0.4

After imputation of SNP array genotyped animals to WGS variants, we obtained 26,447,434 markers on 18 autosomes. In this study, we used HD genotyped crossbred pigs as an intermediate reference. The estimated imputation accuracy (R^2^ reported by Minimac4) is comparable to the cattle study by Daetwyler et al. [2]. The average estimated imputation accuracy for the markers with minor allele frequency (MAF) >= 0.05 reached 0.94. If the markers with estimated imputation accuracy below 0.4 are filtered out, the average estimated imputation accuracy reaches above 0.9 for all MAF classes (Fig. 1). Moreover, as shown in Fig. 1, most of the inaccurate imputed markers (*R*^2^ <= 0.4) are markers with low MAF (0-0.05), which are very challenging to impute accurately due to the lack of haplotype in the reference population. After quality filtering with MAF (0.5%) and Hardy–Weinberg proportions (*p* < 10^-6^), the WGS marker set for association study was 12,596,412 for Duroc, 18,654,181 for Landrace, and 14,522,325 for Yorkshire breeds. As the current computational facility available to us was limited, we could not run GWAS analysis including all animals from a breed, and we therefore adopted the strategy to split each of the breeds into three subpopulations, and then combined results using within-breed meta-analysis.

### Association analysis for average daily gain in Duroc

**Fig. 2:**
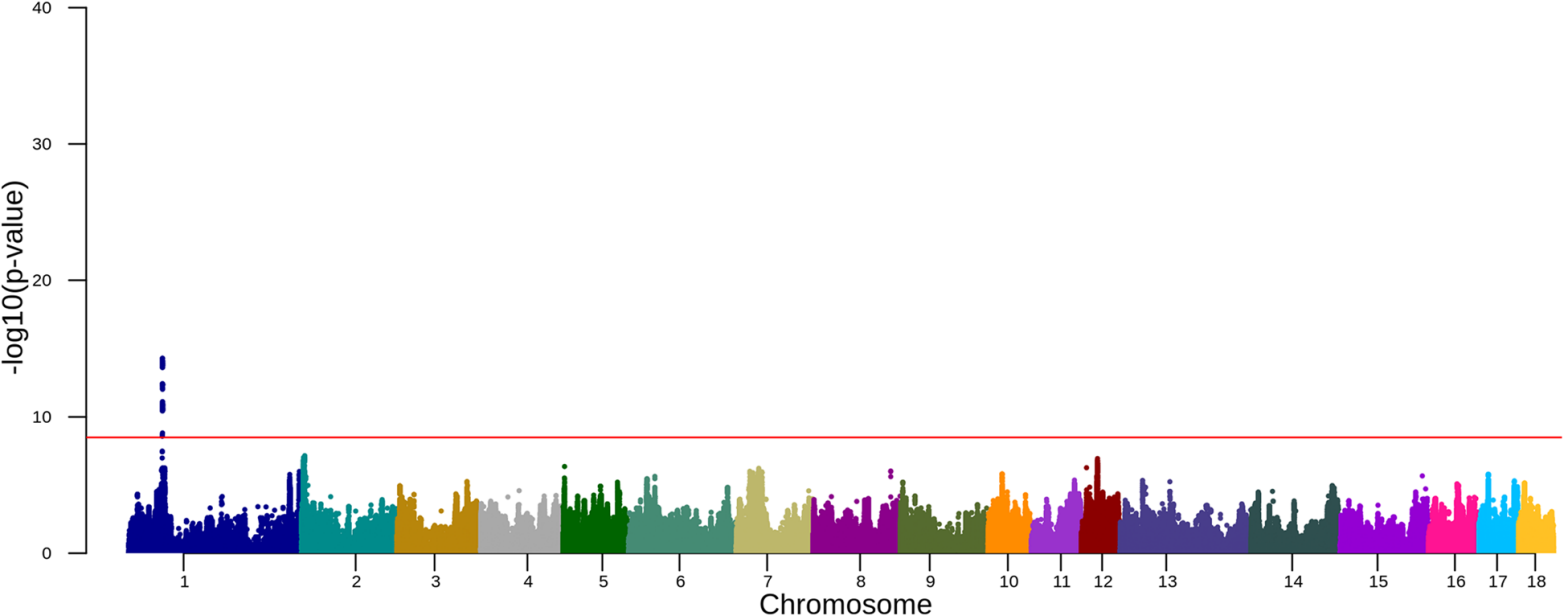
Manhattan plot for the association of SNPs with daily weight gain in Duroc. The red horizontal line indicates a genome-wide significance level [-log10(*p*-value) = 8.5]

In Duroc, we identified one QTL on chromosome 1 in the within-breed meta-analysis (53,054,787-54,008,032; Fig. 2, Supplementary Figs. S1 and S4 and Supplementary Table S1). Splitting into subsets of data based on birth years, we did not identify any QTL in animals born in 2015-2016 and 2017-2018 (Fig. S1a and b). We only detected an association signal in animals born before 2015 (Fig. S1c), and this is the same as in the within-breed meta-analysis (Fig. 2). The lead SNP of this association is 1: 53289914 (rs344908085) with -log_10_(*p*-value) = 14.30. This SNP is located in the intron of the *MRAP2* gene, which encodes Melanocortin-2 receptor accessory protein 2.

### Association analysis of average daily gain in Landrace

**Fig. 3:**
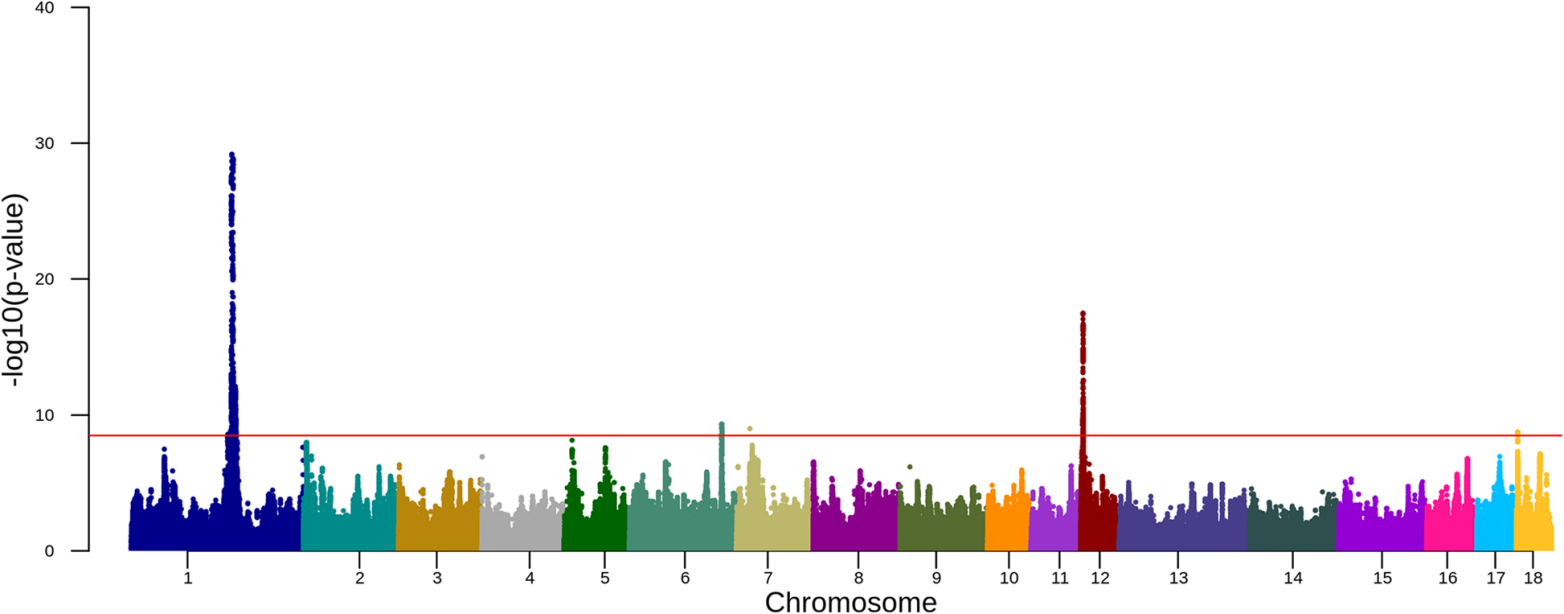
Manhattan plot for the association of SNPs with daily weight gain in Landrace. The red horizontal line indicates a genome-wide significance level [-log10(*p*-value) = 8.5]

In Landrace, we located five QTL on five chromosomes in the within breed meta-analysis (Fig. 3, Table 1, Supplementary Figs. S2 and S5 and Supplementary Table S1). The strongest association signal was 1: 160174493 (rs343467711, -log_10_(*p*-value) = 29.19) with the nearest gene being *CDH20*. The second strongest association signal was on chromosome 12, where the lead SNP was 12: 3639288 (rs1109299516) within the intron of *TK1*. The third-strongest association signal was 6: 146958866 (rs334716220), which was annotated as an intergenic variant with *LEPROT* as the closest gene. On chromosome 7, the lead SNP was 7: 21015982 (rs697892846) near to *ABT1*. On chromosome 18, the lead SNP was 18:2162425. This lead SNP is an intergenic variant with *RNF32* as the nearest gene.

**Table 1:** Genomic regions identified by within-breed meta-analysis from subpopulation genome-wide association analysis of daily weight gain in Landrace

Chr	Lead SNP location (bp)	Region	rs id of lead SNP	-log_10_(*p*-value)	Annotation of lead SNP	Nearest gene
1	160,174,493	159,655,745 ~ 160,704,722	rs343467711	29.19	intergenic_variant	*CDH20*
6	146,958,866	146,482,476 ~ 147,783,245	rs334716220	9.32	intergenic_variant	*LEPROT*
7	21,015,982	20,500,737 ~ 21,587,268	rs697892846	9.01	intergenic_variant	*ABT1*
12	3,639,288	3,530,556 ~ 4,277,570	rs1109299516	17.48	intron_variant	*TK1*
18	2,162,425	1,301,989 ~ 2,856,015	rs793031877	8.74	intergenic_variant	*RNF32*

### Association analysis of average daily gain in Yorkshire

**Fig. 4:**
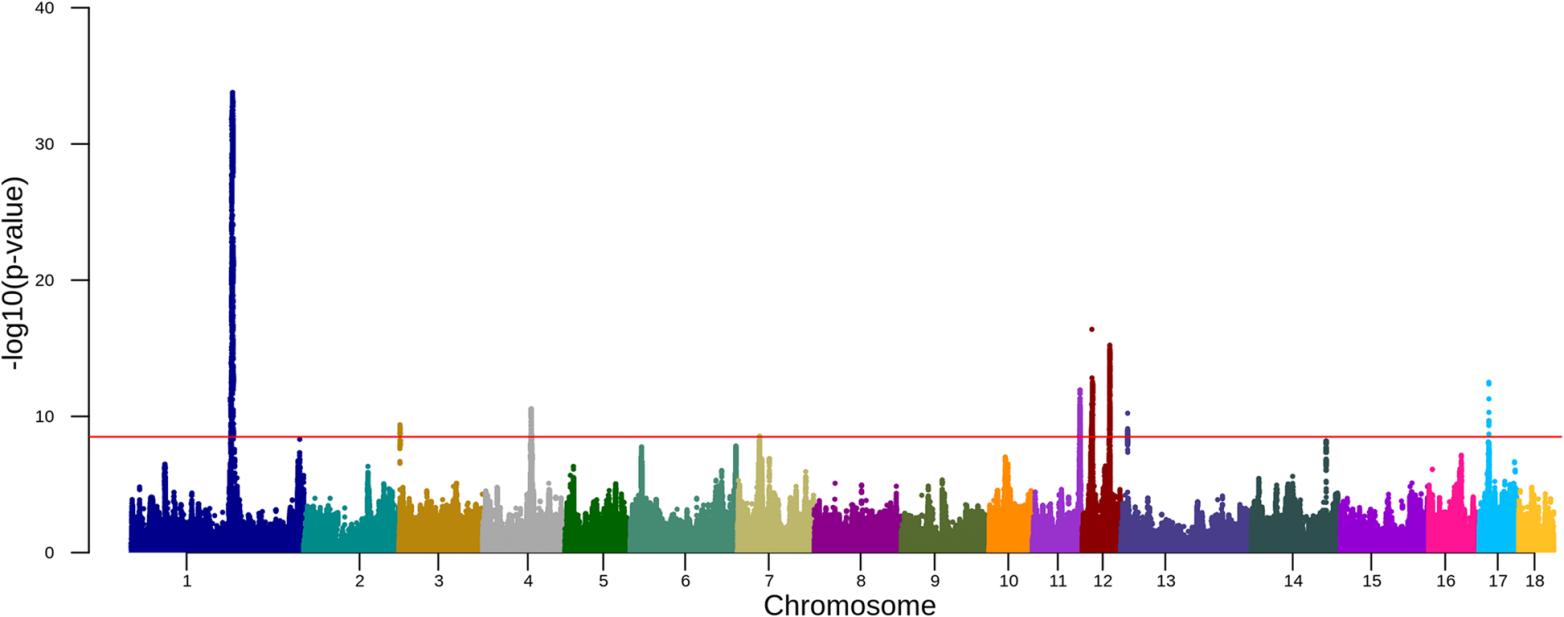
Manhattan plot for the association of SNPs with daily weight gain in Yorkshire. The red horizontal line indicates a genome-wide significance level [-log10(*p*-value) = 8.5]

In Yorkshire, we identified nine QTL on eight different chromosomes in the within breed meta-analysis (Fig. 4, Table 2, Supplementary Figs. S3 and S6 and Supplementary Table S1). The strongest signal was 1:160950166 with -log_10_(*p*-value) equal to 33.78. This lead SNP is an intergenic variant and the nearest gene is *ENSSSCG00000036234*. The QTL interval in Yorkshire largely overlapped with the QTL interval on chromosome 1 of Landrace, however the nearest gene in these two breeds are different. The second-strongest association signal was 12:15311500 located in the intron of *TACO1*. The other lead SNP on chromosome 12 was 12:43812683 with *NF1* as the nearest gene. The fourth-strongest association signal was on chromosome 17 with 17:15758097 (rs694525579) as the lead SNP and located at the intron of *BMP2*. The lead SNP on chromosome 11 was located at 75,538,956 bp (rs319374568) which is within the coding sequence of *LIG4*. On chromosomes 3, 4, 7 and 13, we also found association signals with *ELFN1, RB1CC1, ENSSSCG00000031184*, and *ENSSSCG00000037247* as the nearest genes, respectively (Table 2).

**Table 2:** Genomic regions identified by within-breed meta-analysis from subpopulation genome-wide association analysis of daily weight gain in Yorkshire

Chr	Location of lead SNP (bp)	Region	rs id of lead SNP	-log_10_(*p*-value)	Annotation of the lead SNP	Nearest gene
1	160,950,166	160,704,722 ~ 161,224,815	NA	33.78	intergenic_variant	*ENSSSCG00000036234*
3	1,223,929	1,068,917 ~ 2,206,109	rs343847926	9.38	upstream_gene_variant	*ELFN1*
4	77,498,483	77,174,471 ~ 77,837,179	rs345856128	10.54	intergenic_variant	*RB1CC1*
7	34,791,135	34,405,000 ~ 35,135,343	rs319751202	8.53	intron_variant	*ENSSSCG00000031184*
11	75,538,956	75,215,348 ~ 76,531,480	rs319374568	11.94	synonymous_variant	*LIG4*
12	15,311,500	15,084,562 ~ 15,735,657	NA	16.39	intron_variant	*TACO1*
12	43,812,683	43,485,392 ~ 44,078,471	NA	15.23	intergenic_variant	*NF1*
13	10,845,327	10,754,226 ~ 11,415,685	NA	10.23	intergenic_variant	*ENSSSCG00000037247*
17	15,758,097	15,490,020 ~ 16,171,660	rs694525579	12.50	intron_variant	*BMP2*

### Multi-breed meta-analysis of average daily gain of three breeds

**Fig. 5:**
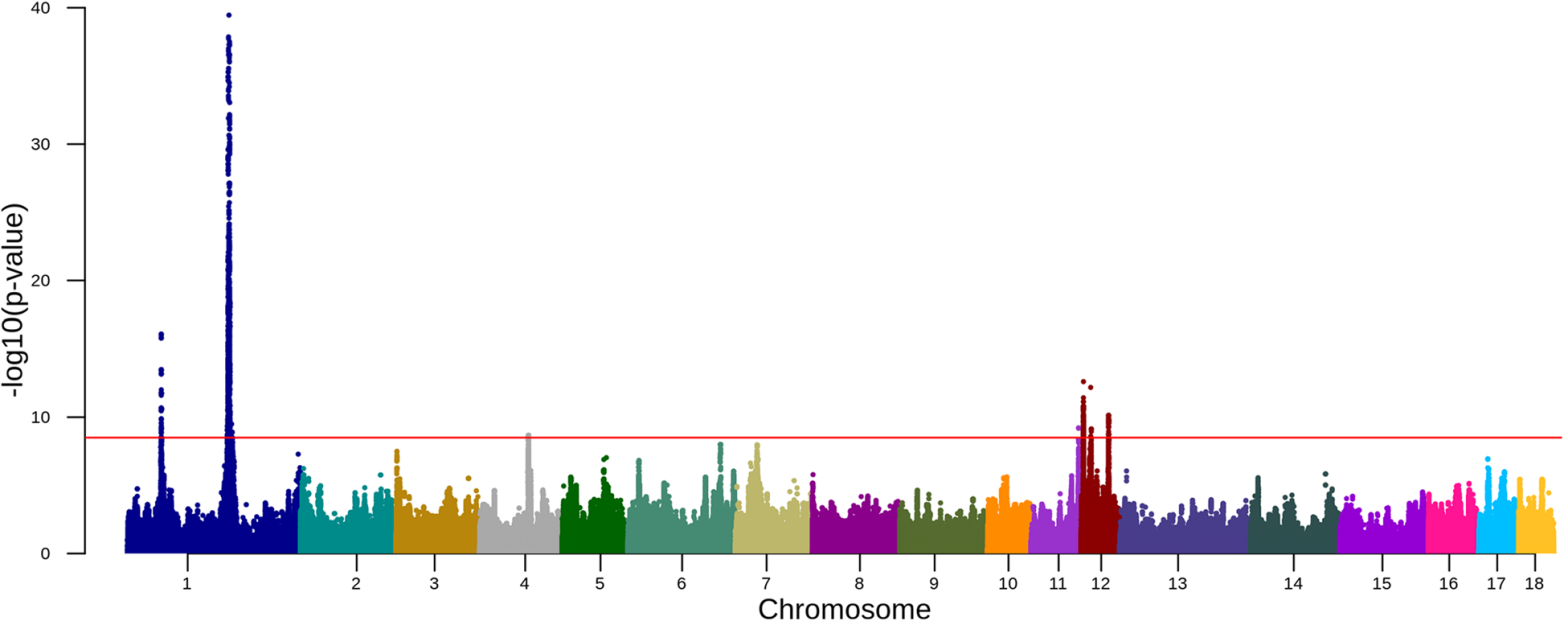
Manhattan plot for the Meta-analysis of SNPs with daily weight gain of three breeds. The red horizontal line indicates a genome-wide significance level [-log_10_(*p*-value) = 8.5]

The total number of QTL detected in multi-breed meta-analysis of three breeds contains 7 QTL on chromosomes 1, 4, 11 and 12 (Fig. 5, Table 3 and Supplementary Table S1). The multi-breed meta-analysis of three breeds did not reveal new QTL compared to the within-breed meta-analysis (Table 3). However, the lead SNP and the detailed QTL interval suggested by the multi-breed meta-analysis of three breeds is slightly different from the within-breed meta-analysis within each breed (Table 3). In the list of the nearest genes from multi-breed meta-analysis, we found some nearest genes are different from the within-breed meta-analysis. On chromosome 1, the multi-breed meta-analysis suggested the nearest gene detected in Yorkshire instead of the one detected in Landrace. On chromosome 11, the multi-breed meta-analysis suggested a new nearest gene *FAM155A* other than *LIG4* suggested from the within-breed meta-analysis of Yorkshire. For the first QTL on chromosome 12, the multi-breed meta-analysis suggested a new nearest gene *TNRC6C* rather than *TK1* suggested from the within-breed meta-analysis of Landrace. And for the third QTL on chromosome 12, the multi-breed meta-analysis also suggested a new nearest gene *WSB1* instead of *NF1* suggested from the within-breed meta-analysis of Yorkshire.

**Table 3:** Genomic regions identified by multi-breed meta-analysis of daily weight gain in three breeds

Chr	Lead SNP location (bp)	Region	rs id of lead SNP	-log_10_(*p*-value)	Annotation of lead SNP	Nearest gene
1	160,827,384	160,137,530 ~ 161,334,328	rs334720929	39.46	intergenic_variant	*ENSSSCG00000036234*
1	53,289,914	53,184,709 ~ 54,008,032	rs344908085	16.09	intron_variant	*MRAP2*
4	77,498,071	77,031,250 ~ 77,782,587	rs345469413	8.66	intergenic_variant	*RB1CC1*
11	75,215,102	74,903,365 ~ 76,080,211	rs338148206	9.20	intron_variant	*FAM155A*
12	3,876,962	3,604,107 ~ 4,277,570	NA	12.61	intron_variant	*TNRC6C*
12	15,311,500	15,084,562 ~ 15,735,657	NA	12.18	intron_variant	*TACO1*
12	43,848,701	43,594,176 ~ 44,823,295	rs324835464	10.13	intergenic_variant	*WSB1*

### Post-GWAS analyses

The candidate gene on chromosome 1 in Duroc and multi-breed meta-analysis of three breeds, *MRAP2*, belongs to the gene ontology (GO) terms “energy reserve metabolic process” (GO: 0006112) and “feeding behavior” (GO: 0007631). In the Mammalian Phenotype database (MPD), multiple phenotype terms are related to obesity or body size for *MRAP2*, e.g., “increased body weight”, “obese”, “increased total body fat amount”, and “increased food intake”. The candidate gene on chromosome 1 in Yorkshire and multi-breed meta-analysis, *ENSSSCG00000036234*, is glutamate decarboxylase 1-like. The GO term showed that this gene responds to the “carboxylic acid metabolic process” (GO: 0019752). *LEPROT*, the candidate gene on chromosome 6 in Landrace, belongs to the GO term “negative regulation of growth hormone receptor signaling pathway” (GO: 0060400). From MPD entry, a mutation in *LEPROT* could cause “increased food intake” or “decreased total body fat amount”. *ELFN1*, the nearest gene for ADG in Yorkshire showed a MPD entry as “increased lean body mass”. *LIG4*, the nearest gene for the lead SNP on chromosome 11 of Yorkshire showed a MPD entry as “decreased body size”. And *BMP2* on chromosome 17 of Yorkshire showed a MPD entry as “decreased body weight”.

We could not established biological support for most of the nearest gene with GO, Kyoto Encyclopedia of Genes and Genomes (KEGG) [28], and MPD. Therefore, we downloaded three RNAseq data sets to find further possible candidate genes (Supplementary Table S2). Although the definition of the trait between GWAS and RNAseq dataset and also the different population architecture could largely reduce the power of this strategy, finding the overlap between GWAS result and RNAseq from different sources could take advantage of the public available dataset to provide biological insight into the GWAS signals. These three data sets included longissimus thoracis muscle tissue [29], liver tissue [30], and longissimus dorsi muscle tissue with liver tissue [31] classifying pigs based on their feed efficiency. We searched for differential expressed genes (DEG) in each QTL region to identify candidate genes with more support from functional activity rather than just picking the nearest gene of the lead SNP (Table 4). For Duroc, no gene within the QTL interval was expressed differently between low and high feed efficiency animals. In Landrace, *H1-*4 located within the QTL region on chromosome 7 and *MNX1* located within the QTL region on chromosome 18 showed differential expression in the liver [31]. The QTL interval at chromosome 1 harbored one DEG between low and high feed efficiency animals, *PMAIP1*. This gene is located in the QTL interval in Yorkshire and multi-breed meta-analysis. In Yorkshire, six other genes in the QTL intervals were DEGs in the liver [31]. They are *PMAIP1*, *RGS20*, *FAM155A*, *ENSSSCG00000017285*, *ENSSSCG00000049912* and *NR1D2*. The QTL interval from multi-breed meta-analysis of three breeds identified four more genes overlapping with the DEGs, *ENSSSCG00000043998, SLC13A2, SEBOX* and *VTN.*

**Table 4:** The differentially expressed genes located within any of the QTL intervals for average daily weight gain in three pig breeds and multi-breed meta-analysis

Gene	log_2_fold change	FDR^a^	Breed
*PMAIP1*	-1.02	4.08e-7	Yorkshire and Meta-analysis
*ENSSSCG00000043998*	-1.02	7.54e-8	Meta-analysis
RGS20	-1.42	2.38e-2	Yorkshire
*H1-4*	-1.37	6.76e-5	Landrace
*FAM155A*	-1.41	1.18e-2	Yorkshire and Meta-analysis
*ENSSSCG00000017285*	-1.64	1.06e-3	Yorkshire and Meta-analysis
*ENSSSCG00000049912*	-1.11	2.15e-3	Yorkshire and Meta-analysis
*SLC13A2*	-1.26	1.98e-2	Meta-analysis
*SEBOX*	1.32	7.92e-4	Meta-analysis
*VTN*	1.19	1.96e-5	Meta-analysis
*NR1D2*	-1.23	3.58e-16	Yorkshire
*MNX1*	1.39	8.74e-3	Landrace

^a^ *FDR* false discovery rate

### Overlap with the associations reported for human growth-related traits

Daily weight gain is a growth-related trait. Therefore, the candidate genes identified for ADG in pigs could overlap with genes in humans for BMI, height, and obesity. If we see homologous genes across mammalian species, that will increase the confidence that the identified candidate genes are true. We checked genes in the QTL interval with BMI (EFO_0004340), height (EFO_0004339), and obesity (EFO_0001073) from the GWAS catalogue [32]. The genes marked as reported genes from the GWAS catalogue that overlapped with genes in our QTL intervals are listed in Table 5. *LEPROT* is the candidate gene suggested by the nearest gene, which is also supported by GO and MPD. This gene is associated with human BMI (Table 5). *PMAIP1* and *FAM155A* are DEGs from the RNAseq data set comparing high and low feed efficiency animals. *PMAIP1* is associated with human height and BMI. *FAM155A* is associated with human height and obesity. *FAM155A* is also the nearest gene in multi-breed meta-analysis. *BMP2* is the nearest gene and showed human BMI and height.

**Table 5:** Human GWAS catalogue for BMI, height, and obesity overlap of genes in QTL intervals for average daily weight gain in three pig breeds and multi-breed meta-analysis

Gene	Human trait	Breed
*MC4R*	BMI, Height and Obesity	Yorkshire and Meta
*PMAIP1*	BMI and Height	Yorkshire and Meta
*IQCE*	Obesity	Yorkshire
*AMZ1*	Height	Yorkshire
*GNA12*	Height	Yorkshire
*LEPR*	BMI and Obesity	Landrace
*LEPROT*	BMI	Landrace
*SLC17A4*	Obesity	Landrace
*SLC17A1*	Obesity	Landrace
*SLC17A2*	Height	Landrace
*SLC17A3*	Obesity	Landrace
*TRIM38*	Height	Landrace
*HIST1H2BD*	Height	Landrace
*BTN1A1*	BMI	Landrace
*ZNF322*	Height	Landrace
*PRSS16*	BMI	Landrace
*U6*	Height	Landrace, Yorkshire and Meta
*DDX42*	BMI	Yorkshire and Meta
*MAP3K3*	Height	Yorkshire and Meta
*THRB*	Height	Yorkshire
*BMP2*	BMI and Height	Yorkshire
*DNAJB6*	BMI	Landrace
*UBE3C*	BMI	Landrace

## Discussion

Since their emergence, GWAS have improved our understanding of the genetic determinants of complex traits of humans and livestock [33]. In human association studies, the availability of large data sets permits GWAS or meta-analysis on a scale of more than 100,000 individuals [34, 35]. However, in livestock, the scale of the sample size for GWAS is usually smaller than 10,000 from a single source of data [4, 5]. Here, we performed GWAS with a total sample size of 201,388 animals from three DanBred commercial pig breeds. With the increase of the sample size applied for GWAS, we have a higher power to detect the genetic variants that contributes to the trait variation.

The imputation of the SNP array genotypes to WGS level in humans [1, 36] has accelerated the association discoveries and unveiled the underlying genetic determinants of complex traits [16, 37–40]. Recently, similar work has also been accomplished in livestock animals, e.g., dairy cattle [2, 11]. However, imputation to WGS has been less widely reported in pigs. Most of the work has focused on evaluating the power to impute low-density marker sets (10k, 9k, etc.) to medium-density marker sets (60k) and achieved an imputation accuracy above 0.95 [41–44]. In 2019, van den Berg et al. [45] used two-step strategies (first 80 to 660k, and then to WGS) similar to the strategy followed in the current study. However, the average estimated imputation accuracy for all WGS variants was only around 0.5 for Large White and around 0.4 for Dutch Landrace [45]. In our study, we achieved a much higher estimated imputation accuracy (Fig. 1), which is comparable to that in cattle [2].

In cattle, it is well supported that a multi-breed reference population can achieve higher imputation accuracy [11]. The same strategy has also been applied in pig imputation [45]. Unlike dairy cattle where most animals are purebred, most pigs in the production are two-way crossbred sows or three-way crossbred slaughter animals. Previous work has shown that imputation of a low-density marker set of crossbred to a high-density marker set using a purebred reference population or imputation of a low-density marker set of purebred to a high-density marker set using a crossbred reference population both resulted in high accuracy [41, 43]. In this study, we tested whether the HD genotypes available from three-way crosses could be used as an intermediate reference panel for imputation from low-density genotyped animals to WGS level to achieve higher accuracy. The results confirmed that the HD crossbred genotypes could be used as an intermediate reference panel for purebreds to impute to WGS variants level.

In this study, we reported the estimated imputation accuracy from Minimac4 instead of calculation of the empirical imputation accuracy. Previous study have shown that the R-sq values estimated by Minimac3 (same as Minimac4) were highly correlated with correlation-based empirical measures [46, 47]. Of course, using the estimated accuracy could limit the direct comparison between studies. However, as imputation becomes a routine work for research groups, scientists are aware of the differences between these two accuracy parameters. Besides this, low imputation accuracy may increase the false negative rate in GWAS, but unlikely to increase false positive rate. Furthermore, the estimated accuracy is sufficient for us to filter out the low quality imputed markers. Therefore, we decided to report the estimated imputation accuracy.

RNA-seq is a powerful tool to carry out functional studies. However, in livestock studies, scientists face the difficulty to choose the right tissue and right development stage related to traits. Previous reports in human found that the estimated correlation of genetic effects of cis-eQTLs between blood tissue and brain tissue could be high as 0.70 [48]. Of course, in such human studies, we could lose some tissue-specific expression genes. Meanwhile, without using the same samples for a GWAS study and a RNA-seq study, it is still worth to combine two types of data [49]. In the above mentioned two human studies, they showed the possibility of using RNA-seq data which are different from the mapping population and with an inconsistency of the study traits and tissues. So, using related-traits RNA-seq data, which are from different breeds could still facilitate finding the common underlying genetic factors between related traits. Therefore, in this study, we used three datasets comparing the DEGs between high and low feed efficiency to prioritize our GWAS result. There was a risk that the difference of the segregation of alleles in different breeds, and the difference between feed efficiency and ADG traits could results in no additional information on candidate genes. Only one of the three RNA-seq datasets showed common genes with our GWAS results.

The GWAS results for three breeds are quite different. The majority of the differences comes from the segregation of different alleles in three breeds, which could be part of the consequence of selection. In the Danbred system, the breeding goal of Duroc is different from the common breeding goal of Landrace and Yorkshire: The goal for Duroc pigs has more weight on growth, leanness, and feed efficiency, whereas the goal for Landrace and Yorkshire has more weight on maternal traits [50]. So certain loci underlying growth may be fixed in Duroc and still segregating in Yorkshire and Landrace. This could be part of the reason why we observed different mapping result between the three breeds. Meanwhile, we also observed differences of the GWAS results between subpopulations. There are two major reason for this: 1) these three breeds are under intensive selection; 2) the splitting of the population could reduce the mapping power.

Daily weight gain is a key trait in pig breeding goals since it plays an important role in economic return. The QTL mapped for daily weight gain in pigs was spread across all chromosomes [51]. With the large sample size, we had a high power to locate the QTL. We checked the overlap of the genes in the QTL intervals for daily weight gain pigs with an association reported for three human growth-related traits (human height, obesity, and BMI) from the GWAS catalogue [32]. The underlying logic for this strategy was threefold: 1) the similarity of genetic determinants in pigs and humans; pigs are used as model species for human biomedical research [52]; 2) the share of the causal genes may generate new knowledge about gene function [7]; 3) the nearest genes of the lead SNP from GWAS may not be the causal ones. Furthermore, the choice of the human traits was based on two criteria, 1) traits are growth related; 2) traits should capture the common causal genes, and so we included child growth trait for these three human traits. Finally, we found at least one gene for each QTL interval for ADG in pigs overlapping with the reported genes for human growth-related traits.

Human height, BMI and obesity are classical complex traits in human genetics. The accumulation of the knowledge about these trait makes them as a gold mine to understand mammalian growth related traits. By comparing our result with these three traits, we found some genes that are the nearest genes from the GWAS and one gene in the DEGs list. *LEPROT* is the nearest to the lead SNP on chromosome 6 in Landrace. The GO and MPD have an entry to support that this gene is related to mammalian growth. In a human study, GWAS of 7,215 children revealed that *LEPROT* is one of the important loci for early growth [53]. *FAM155A* is the nearest gene from GWAS of Yorkshire which is also supported by DEGs. This gene was reported in a study for human height, BMI and obesity [54–57]. *PMAIP1* is located in the QTL interval, showed in the DEGs list and associated with human BMI and height [58, 59]. *BMP2* is the nearest gene and literature showed association with human height and BMI [60, 61]. Besides, *MRAP2* is an important candidate for ADG in pigs since it has support for GO annotation and Mammalian Phenotype database. *ENSSSCG00000036234, ELFN1, LIG4* could also be good candidate genes with support from nearest gene and GO annotation.

## Conclusions

In this study, for three large pig populations, we have accurately imputed from low-density chip to WGS with a high estimated imputation accuracy. This is useful for deciding on an imputation strategy in future genomics studies in pigs. The validation of the QTL interval with the GWAS catalogue of human height, obesity, and BMI showed that GWAS accurately map the QTL region and suggested several candidate genes for daily weight gain in pigs. Our results will improve our understanding of genetic architecture of ADG in pigs and can also be exploited in pig breeding to improve daily weight gain.

## Methods

### Animals and phenotype

Phenotypic records and SNP array genotypes from three DanBred pig breeds, Duroc, Landrace, and Yorkshire, were provided by SEGES – Breeding & Genetics in pigs. Corrected phenotypes for ADG from 30-100 kg were computed using predicted effects from the routine genetic evaluation model. The corrected phenotype of ADG for an individual equals the sum of the predicted breeding value and predicted residual, or in other words the phenotype minus the sum of all predicted non-genetic fixed and random effects.

Briefly, the routine genetic evaluation model is a four-variate model with traits: average daily gain 7-30 kg, average daily gain 30-100 kg, meat percentage computed from the scanning of back fat and weight at the time of scanning, and feed efficiency. Fixed effects are year-herd-month (all traits), sex (except for feed efficiency, since only boars have that measurement), and start weight (except for meat percentage). Random effects are breeding value (all traits), pen (ADG, meat percentage), and litter (all traits).

### Genotyping and whole-genome resequencing

In this study, we used three sets of genotypes, starting from low- to medium-density SNP array through to the whole-genome variant level.

#### Low- to medium-density SNP genotyping

In total, 201,388 pigs were genotyped with multiple low- to medium-density (8.5 to 70k) SNP chips. These were 42,790 Duroc, 88,984 Landrace, and 69,606 Yorkshire. The number of Duroc, Landrace, and Yorkshire animals genotyped with a Genomic Profiler (GGP) Porcine LD array (8.5k) chip was 7,328, 13,238, and 13,282, respectively; and the number of pigs genotyped with a GGP_HD_Porcine chip (43k) was 31,287, 68,800, and 49,313, respectively. The number of pigs genotyped with an Illumina PorcineSNP60 BeadChip (60k) or GGP Porcine HD array (70k) was 4,175 Duroc, 6,946 Landrace, and 7,011 Yorkshire.

#### High-density (HD) SNP array

We used high-density genotypes using Affymetrix Axiom PigHD SNP chips (Axiom_PigHDv1, 658k) of 474 three-way crossbred pigs as the intermediate reference panel. The animals were part of the “MetaPig – Modulation of the pig gut metagenome to increase feed efficiency” project (http://www.metapig.eu). These three-way crossbreds are produced by crossing F1 sows from Landrace and Yorkshire inseminated with mixed semen from Duroc boars. The details on this HD genotype data set are presented by Cai et al. [50].

#### Whole-genome resequencing

A total of 217 animals of three DanBred commercial pig breeds, i.e., 89 Duroc, 61 Landrace, and 67 Yorkshire, were sequenced. The animals for sequencing were selected based on their genetic contribution to the genotyped animals born in 2010, 2011, and 2012. The detail of the sequencing and processing of the data can be found in our previous study [50]. For each individual, paired-end read trimming was performed using trim-fastq from the PoPoolation package [62]. Filtered reads were aligned to the porcine reference genome build 11 [63] by the Burrows-Wheeler Aligner (BWA version 0.7.17) [64], employing “bwa-mem”. SAMtools version 1.8 (Li et al. 2009) was used for sorting, merging, and marking potential PCR duplicates. From here until the VCF file, the reads were processed using the Genome Analysis Toolkit (GATK version 3.8) [65] according to the 1000 bull genome project pipeline [2].

We applied hard filtering as following. For SNPs, we applied: "QD < 2.0, "SOR > 3.0", "FS > 60.0", "MQ < 40.0", "MQRankSum < -12.5", “ReadPosRankSum < -8.0", and “DP < 4 || DP > 6600". For INDELs, we applied "QD < 2.0", "FS > 200.0", "ReadPosRankSum < -20.0", "InbreedingCoeff < -0.8","DP < 4 || DP > 6600", and "SOR > 10.0". Then we combined the filtered SNP set and INDEL set as the final reference panel.

#### SNP map position

The probe sequence (50 bp flanking the SNP) of the 10k, 50k, 60k, and HD chip array was mapped to the *sus11.1* assembly by bwa-mem [64]. Only the probes mapped uniquely and CIGAR string with “50M” were retained for the following imputation. We replaced the location of SNPs with the location of the mapping result.

### Genotype imputation

The genotypes (10, 43, 60 and 70 k) of pigs from all three breeds were combined and phased using Eagle [66]. The HD and WGS marker sets were phased following the procedure of Mesbah-Uddin et al. [67]. Briefly, the genotypes were phased by Beagle4 (r1274) [68] to calculate the genotype probability, and then SHAPEIT2 (v2.r837) [69] was used to call the genotype of the markers with a genotype probability less than 0.99. For imputation of the combined data set to the WGS level, we adopted two-step imputation strategies described in van Binsbergen et al. and Brøndum et al. [10, 11]. In the first step, we imputed all individuals genotyped by low- and medium-density chips to HD level using the 474 three-way crossbred animals as an intermediate reference panel using Minimac4 [70]. In the second step, we imputed this imputed HD marker set to whole-genome sequencing (WGS) level using the 217 WGS individuals using Minimac4 [70]. Before GWAS, we filtered away all SNPs with minor allele frequency below 0.5%, with a large deviation from Hardy–Weinberg proportions (*p* < 1.0^− 6^), or an *R*^2^ value of the estimated imputation accuracy estimated by Minimac4 of less than 0.4.

### Association analysis and meta-analysis

Due to the large number of animals in each breed, the GWAS by GCTA required a huge amount of computer memory. To deal with this issue, we separated the population into three similar-size subsets based on the birth year (Table 6). Then we run sub-population GWAS in each subset followed by within-breed meta-analysis to combine results from subsets for a breed. For sub-population GWAS, we estimated the genomic relation matrix (GRM) for all autosomes by GCTA [71] using the imputed HD marker set. The method that was used to estimate GRM between individuals using SNP data is implemented in GCTA [71]. Briefly, genotype dosages and allele frequency of each SNP between one pair of individuals *i* and j were used to calculate the relationship score, then average relationship score across all SNPs was calculated as the relationship between individuals *i* and j. We ran the association study for each chromosome with the GRM obtained above using GCTA [71] in each subset, using a mixed-model approach, *GCTA-MLMA* using the following model:

$$y=a+ bx+g+e$$

**Table 6:** The number of animals, genomic inflation factor (lambda), and the ratio between additive variance (VA) and phenotypic variance (VP), i.e., genomic heritability for each subset of data for three pig breeds

	Duroc			Landrace			Yorkshire		
	Before 2015	2015-16	2017-18	Before 2015	2015-16	2017-18	Before 2015	2015-16	2017-18
Number	15,190	12,810	14,663	22,121	18,960	46,593	22,179	16,225	29,840
Lambda	0.95	0.95	1.00	0.98	0.93	0.97	0.92	0.92	0.92
Lambda	1.32			1.30			1.35		
${{V}_A}\!\left/ {{V}_P}\right.$	0.252	0.149	0.175	0.282	0.299	0.228	0.282	0.250	0.230

where *y* is the phenotype value, *a* is the population mean, *b* is the fixed effect the candidate SNP to be tested for association, *x* is the SNP genotype, and g is the polygenic effect captured by the GRM calculated using the imputed HD marker set and *e* is the residual. We set the genome-wide significant threshold as -log_10_ (*p*-value) > 8.5 with Bonferroni correction (0.05/13,000,000). Then we performed within-breed meta-analysis using METAL [72] with the option of genomic control to deal with the inflation. The number of animals with both genotype and phenotype information was 198,623 (42,663 Duroc, 87,674 Landrace, and 68,244 Yorkshire). At last, we performed multi-breed meta-analysis to investigate the association signals across the three breeds with the same parameter as within-breed meta-analysis. The details of the animal number of each breed, the animal number of each subpopulation, and the genomic inflation factor (lambda) estimated by METAL [72] are listed in Table 6.

### Post-GWAS analysis

The location of the annotated genomic feature was extracted from Ensembl [73]. The GO and KEGG pathway annotation of pig genes were also extracted from Ensembl [73]. We performed the variants annotation using the Ensembl Variant Effect Predictor (ver99) [74]. The possible consequence of the impact of the variant on the phenotype based on mouse mutation lines was retrieved from the Mammalian Phenotype database (MPD) [19].

### Differentially expressed gene analysis

We downloaded three RNA-seq dataset to perform the differentially expressed gene (DEG) analysis: PRJEB23668, PRJEB29969 and PRJEB23289. PRJEB23668 included liver tissue from 20 Maxgro (Hermitage Genetics) x (German Landrace x Large White) pigs. PRJEB29969 included 96 samples from (Large White x Landrace) x Meatline liver or longissimus dorsi muscle tissue. PRJEB23289 included longissimus thoracis muscle tissue from 20 Maxgro (Hermitage Genetics) x (German Landrace x Large White) pigs. Each of the three data sets were divided into high feed efficiency and low feed efficiency groups.

To use the new assembly and annotation information on pigs, we decided to reanalyze three previously reported RNA-seq data from the liver and/or muscle tissue between high and low feed efficiency animals [29–31]. The raw reads were downloaded from ENA (https://www.ebi.ac.uk/eva). We used Trimmomatic (Ver 0.39) [75] to remove potential adapter sequence and trim low-quality reads. For DEG analysis, we downloaded the pig reference genome and transcriptome from Ensembl (v99) [73]. We built a decoy-aware transcriptome index file with the genome sequence and transcriptome sequence following the guidance of Salmon (v1.2.0) [76]. The final clean data were mapped to the decoy transcriptome using Salmon (v1.2.0) [76]. The DEG analysis was performed using DESeq2 [77]. The genes with an adjusted *p* value < 0.05 and log_2_ fold change > 1 or < -1 were regarded as DEGs.

### Validation with GWAS catalogue of human height, obesity, and body mass index

The GWAS catalogue of human height (EFO_0004339-withChildTraits_2020_06_10), obesity (EFO_0001073-withChildTraits_2020_06_10), and body mass index (BMI, EFO_0004340-withChildTraits_2020_06_04) was downloaded from the NHGRI-EBI Catalog of human genome-wide association studies [32]. The selection of the human traits was limited to growth related. We would like to find the common genetic factors that underlies human height, obesity, BMI and pig ADG on individuals in their growing period. We checked the overlap of the genes in the QTL intervals from our analysis in pigs with the reported genes from the GWAS catalogue in humans.

## Supplementary Information

**Additional file 1: Supplementary Figure S1-6.**

**Additional file 2: Supplementary Table S1.**

**Additional file 3: Supplementary Table S2.**

## Data Availability

Genome assembly data used in this study were obtained from the NCBI (https://ftp.ncbi.nlm.nih.gov/genomes/all/GCF/000/003/025/GCF_000003025.6_Sscrofa11.1/). All annotation information was obtained from a publicly available source (http://www.ensembl.org). Whole-genome sequences and individual SNP genotype data in this study are available only upon agreement with the breeding organization and should be requested directly from the authors. RNA-seq data were from bio project PRJEB23289, PRJEB23668, and PRJEB29969. The genotype and phenotype used and/or analysed during the current study available are available from the authors with the permission of SEGES Danish Pig Research Centre (https://pigresearchcentre.dk/). Access to these data for research requires permission from DataGene under a Data Use Agreement.

## Abbreviations

QTL: Quantitative trait loci
GWAS: Genome-wide association study
ADG: Average daily gain
WGS: Whole genome sequencing
HD: High density
MAF: Minor allele frequency
SNP: Single nucleotide polymorphism
GO: Gene ontology
KEGG: Kyoto Encyclopedia of Genes and Genomes
