# Supplementary material for: Large-scale association study on daily weight gain in pigs reveals overlap of genetic factors for growth in humans
Source: BMC Genomics. 2022 Feb 15;23:133. doi: 10.1186/s12864-022-08373-3 (PMC8845347; doi:10.1186/s12864-022-08373-3)
Supplement: Supplementary file 1 — Additional file 1: Supplementary Figure S1-6. [file 12864_2022_8373_MOESM1_ESM.docx]

**Large-scale Association Study on Daily Weight Gain in Pigs Reveals Overlap of Genetic Factors for Growth in Humans**

Zexi Cai, Ole Fredslund Christensen, Mogens Sandø Lund, Tage Ostersen, Goutam Sahana


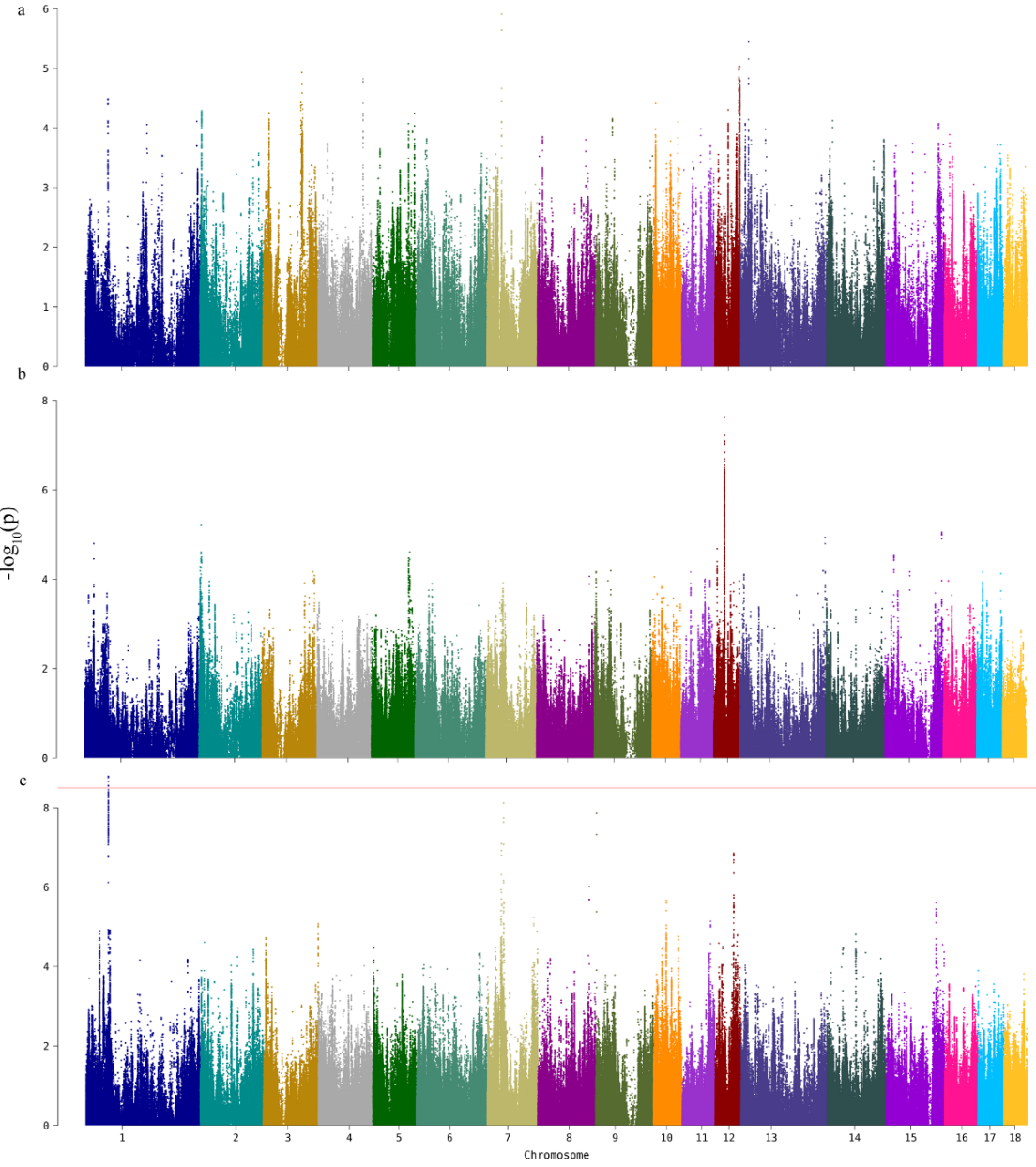


Figure S1. Manhattan plot for association of SNPs with average daily gain of Duroc in each sub-population. Red horizontal line indicates genome-wide significance level [−log10(P) = 8.5]. (a) Y1516 sub-population; (b)Y1718 sub-population; (c) Before-15 sub-population.


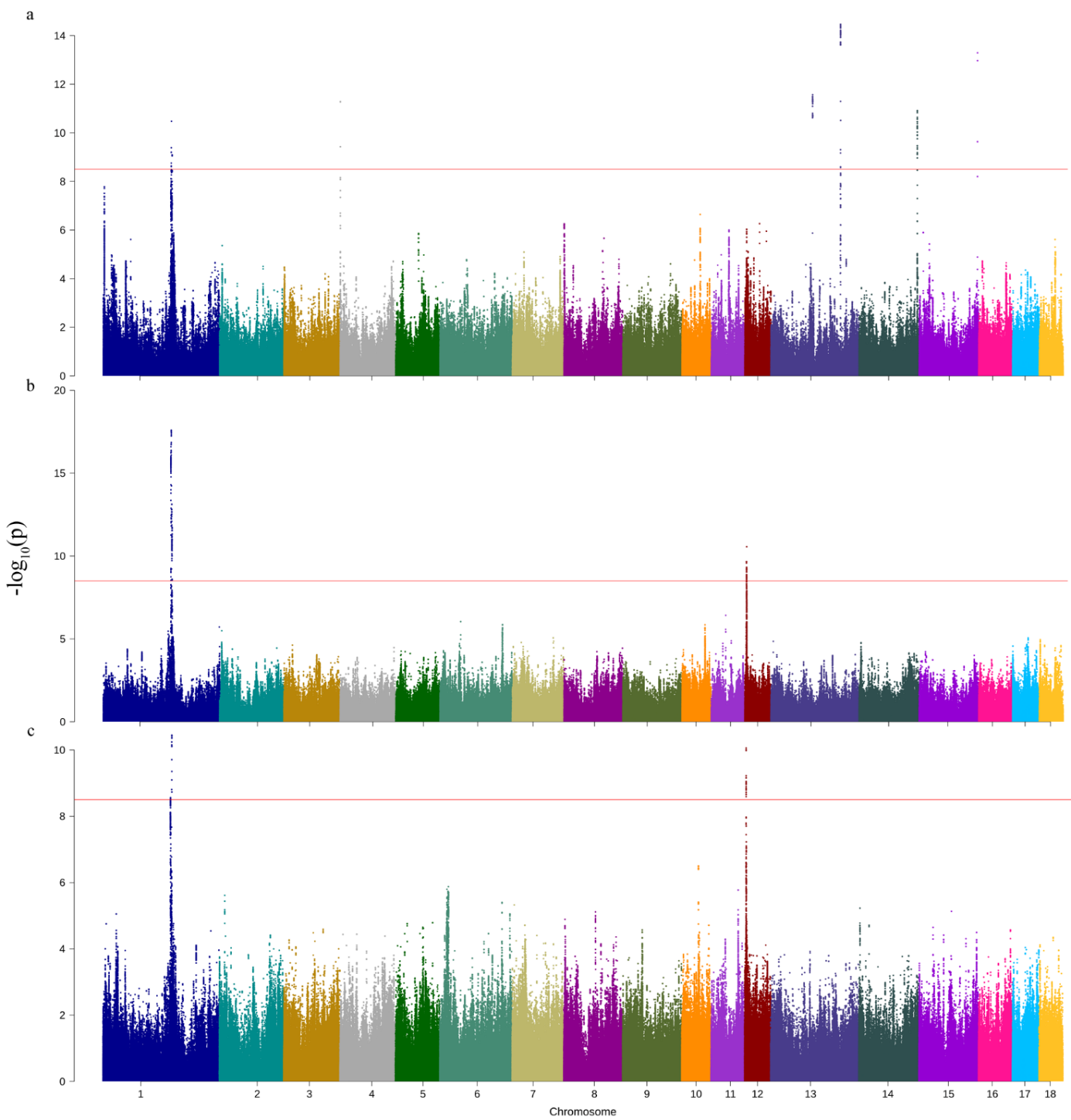


Figure S2. Manhattan plot for association of SNPs with average daily gain of Landrace in each sub-population. Red horizontal line indicates genome-wide significance level [−log10(P) = 8.5]. (a) Y1516 sub-population; (b)Y1718 sub-population;(c) Before-15 sub-population.


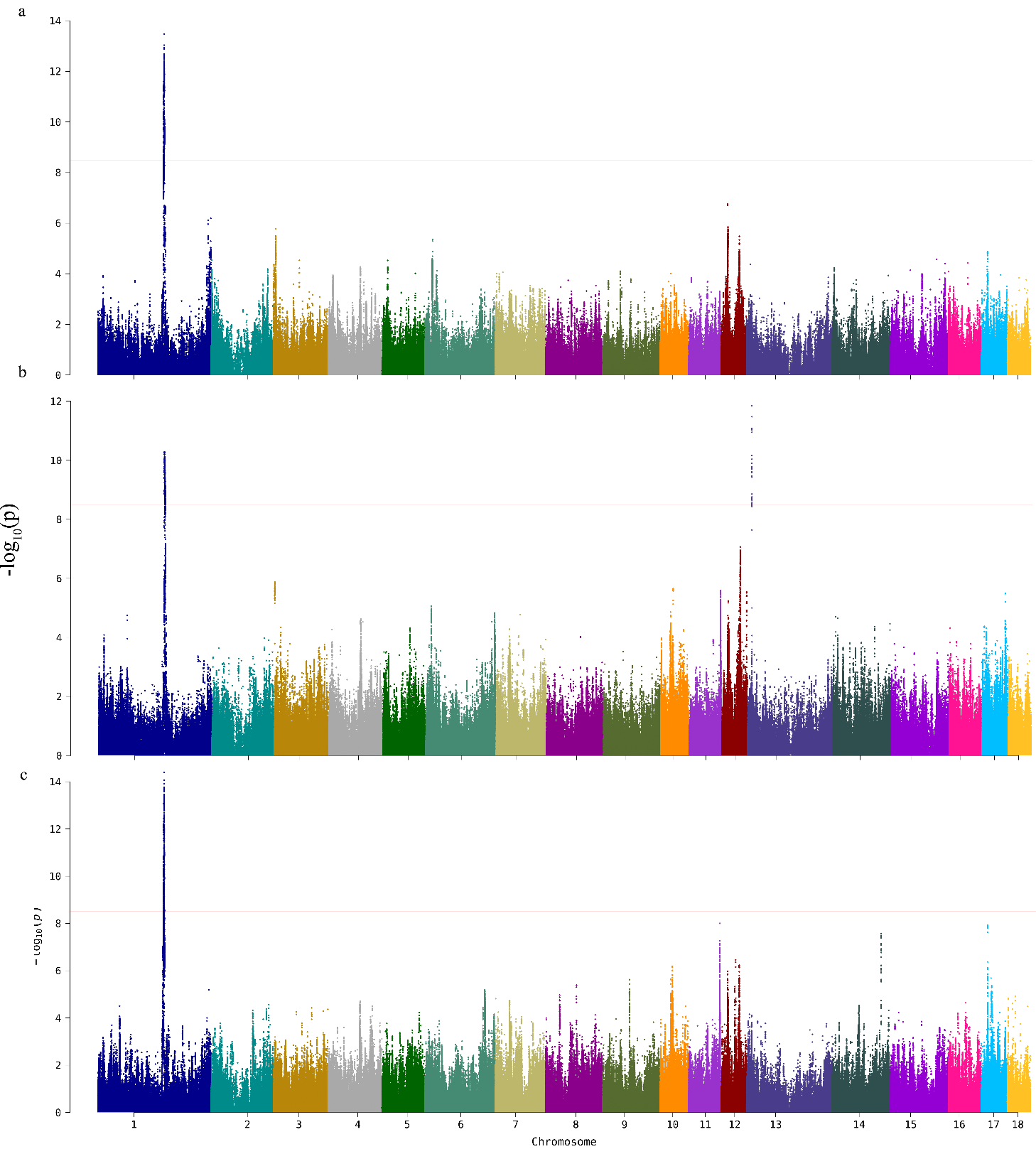


Figure S3. Manhattan plot for association of SNPs with average daily gain of Yorkshire in each sub-population. Red horizontal line indicates genome-wide significance level [−log10(P) = 8.5]. (a) Y1516 sub-population; (b)Y1718 sub-population;(c) Before-15 sub-population.


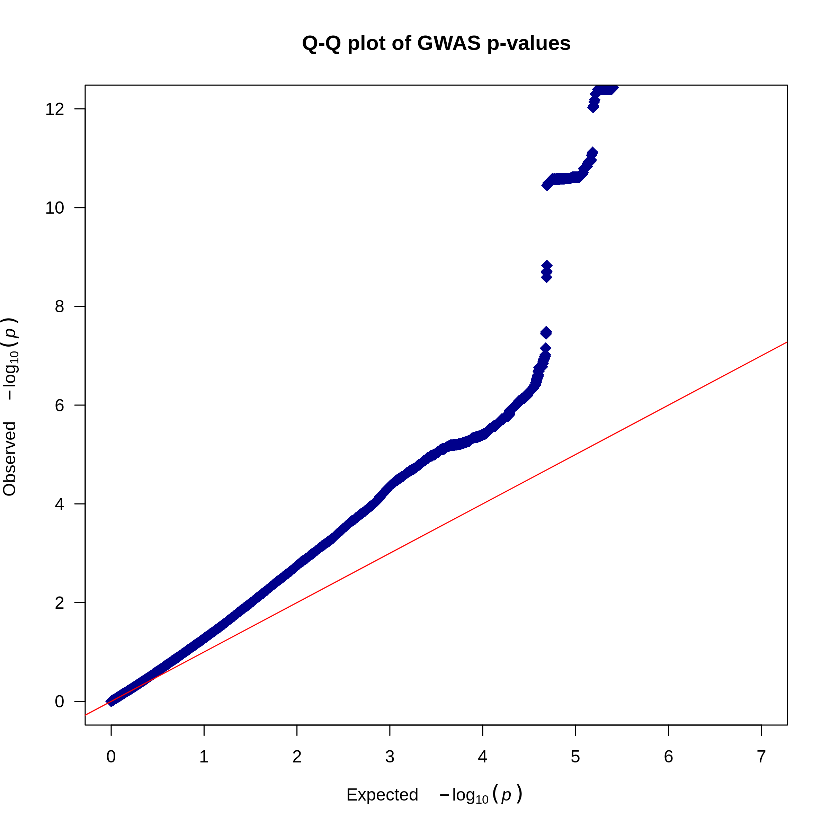


Figure S4. Quantile-quantile plot for association of SNPs with average daily gain of Duroc.


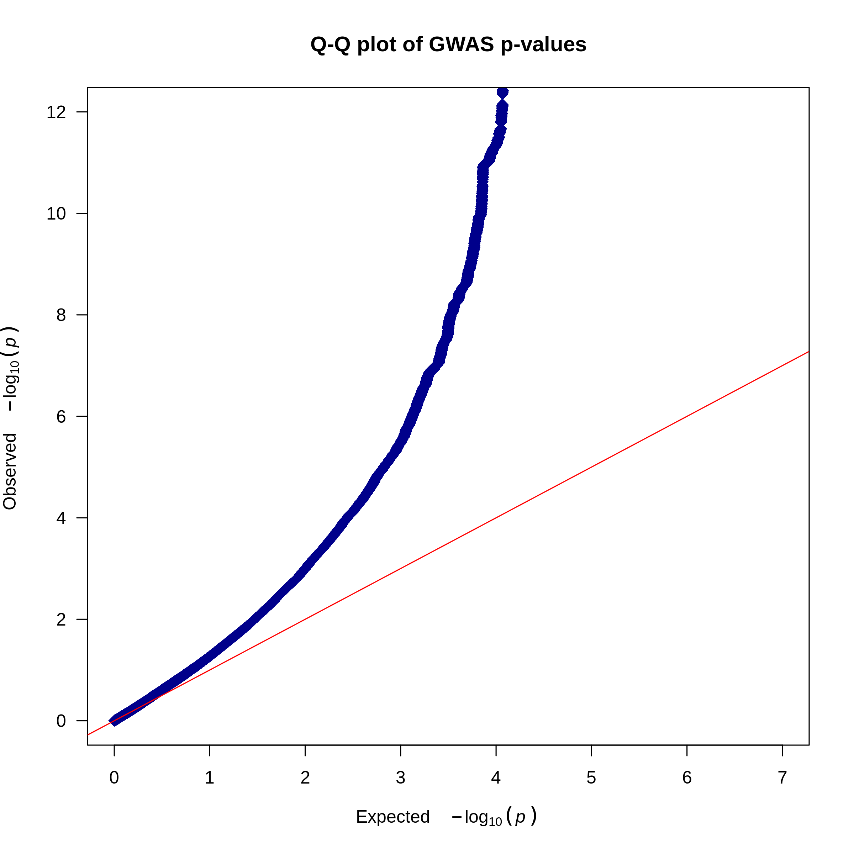


Figure S5. Quantile-quantile plot for association of SNPs with average daily gain of Landrace.


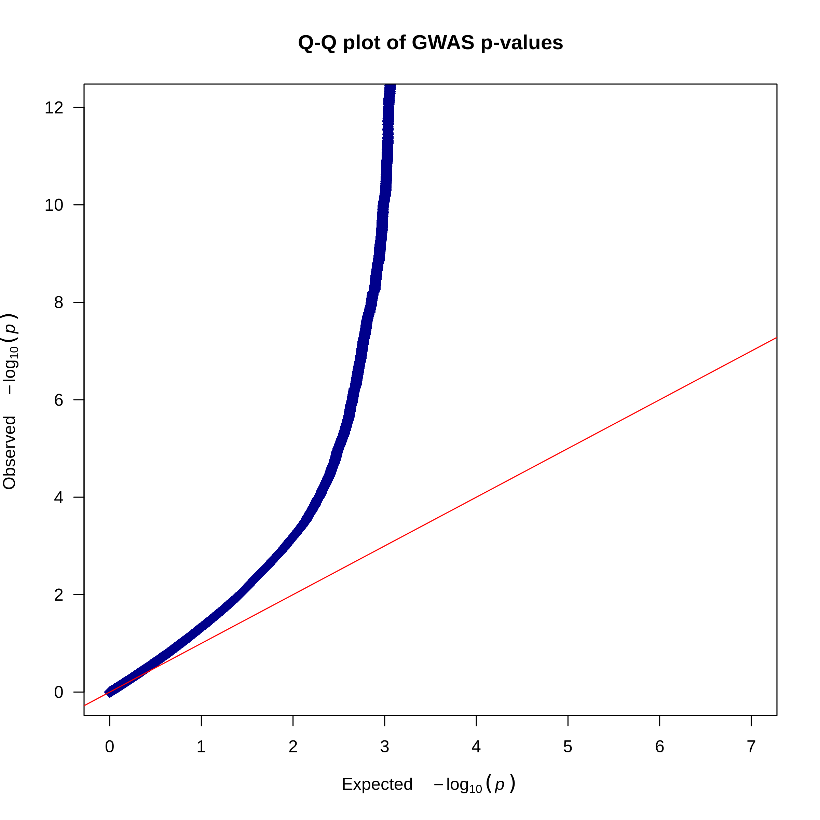


Figure S6. Quantile-quantile plot for association of SNPs with average daily gain of Yorkshire.
